# *Torreya nucifera* seed oil improves 3T3-L1 adipocyte differentiation

**DOI:** 10.1186/s12906-021-03429-5

**Published:** 2021-10-07

**Authors:** Eunbi Koh, Boram Kim, Kyungoh Choi

**Affiliations:** Department of Chemical and Material Engineering, The University of Suwon, 17, Wauan-gil, Bongdam-eup, Hwaseong-si, Gyeonggi-do 18323 Republic of Korea

**Keywords:** *Torreya nucifera* seed oil, Adipocyte, Differentiation, PPARγ, STAT3

## Abstract

**Background:**

Adipose tissue is a critical regulator of lipid storage and endocrine function. Impairment of the recruitment of new adipocytes in the adipose tissue is associated with ectopic fat accumulation, diabetes and insulin resistance. *Torreya nucifera*, an evergreen conifer that grows in warm temperate climates, has been found to exert beneficial effects against inflammation, infection and diabetes. However, the molecular mechanisms responsible for these effects at the cellular level remain unknown. This study aimed to investigate effects of *Torreya nucifera* seed oil (TNSO) on 3T3-L1 adipocyte differentiation and its underlying regulatory mechanism.

**Methods:**

To investigate the effects of TNSO on adipocyte differentiation, 3T3-L1 cells were induced to differentiate for 5 days in the presence of 0.75 μL/mL TNSO. Oil Red O staining and an assay for intracellular triglyceride were performed to determine the extent of lipid accumulation in 3T3-L1 cells. To elucidate the underlying mechanism of TNSO, adipogenic gene expression was analyzed using quantitative real-time PCR. Moreover, we monitored TNSO-derived activation of PPARγ and STAT3 with 3T3-L1 reporter cell lines engineered to secrete *Gaussia* luciferase upon the interaction of a transcription factor to its DNA binding element.

**Results:**

Oil Red O staining revealed that TNSO improved the differentiation of 3T3-L1 preadipocytes into mature adipocytes. The mRNA levels of adipogenic genes, including adiponectin, fatty acid synthase (FAS) and adipocyte fatty acid-binding protein (FABP4), were upregulated and intracellular triglyceride levels increased upon TNSO treatment. We also established that adipocyte differentiation was improved by TNSO-derived activation of PPARγ and STAT3.

**Conclusions:**

Our results suggest that TNSO improves adipocyte differentiation by regulating the activation of adipogenic transcription factors, indicating that it may serve as a potential treatment strategy for adipocyte dysfunction.

**Supplementary Information:**

The online version contains supplementary material available at 10.1186/s12906-021-03429-5.

## Background

Adipose tissue regulates energy storage and energy supply in the body. Healthy adipose tissue relies on a steady renewal of adipocytes, which differentiate in a multistep process called adipogenesis. Adipogenesis is crucial for metabolic homeostasis and impairment of adipogenesis is a prominent characteristic of obesity and insulin resistance [1]. In particular, during aging, physiological reactive oxygen species (ROS) levels are altered and conventional adipogenesis is disrupted. The suppression of adipocyte renewal via oxidative stress increases the risk of developing hypertrophic adipocytes, lipotoxicity and insulin resistance [2].

Adipocyte differentiation is a complex process involving several genes, whose expression is regulated through various adipocyte-specific transcription factors [3, 4]. A key transcription factor in the adipocyte differentiation program is peroxisome proliferator-activated receptor gamma (PPARγ) and signal transducer and activator of transcription 3 (STAT3) also plays a critical role in the regulation of other adipocyte-specific transcription factors [5, 6]. The degree of activation of these transcription factors is associated with the expression of adipocyte-specific genes including fatty acid synthase (FAS), adipocyte fatty acid-binding protein (FABP4) and adiponectin, which regulate metabolic processes in adipocytes [7–9].

*Torreya nucifera* is an evergreen conifer growing in Korea, China, and Southern Japan. According to previous reports, *Torreya nucifera* has been widely used as a traditional medicine owing to its beneficial effects on helminth infestation, constipation, diabetes mellitus, and hemorrhoids [10, 11]. Previous studies on *Torreya nucifera* primarily focused on the physicochemical properties of extracts from its leaves, fruits, and wood. Yoon et al. showed that essential oils drived from *Torreya nucifera* leaves suppressed growth of skin pathogens and secretion of inflammatory substances by macrophage cells [12]. An ethyl acetate fraction prepared from the seed of *Torreya nucifera* exhibited anti-inflammatory activities in macrophage cells [11]. In addition, the ethanol extract of *Torreya nucifera* leaves exhibited inhibitory activity on the main protease of coronavirus that causes severe acute respiratory syndrome [13]. Although *Torreya nucifera* has been widely used traditionally, its effects on adipocyte differentiation and the underlying mechanism at the cellular level have not previously been studied.

In this study, we investigated the effects of *Torreya nucifera* seed oil (TNSO) on 3T3-L1 adipocyte differentiation. Adipocyte differentiation was assessed using Oil Red O staining, measurement of intracellular triglyceride levels, and quantitative real-time PCR which was performed to analyze adipogenic gene expression. Furthermore, to elucidate the mechanism underlying the effects of TNSO, a change of PPARγ and STAT3 activation was monitored using 3T3-L1 reporter cell lines.

## Methods

### Chemicals

*Torreya nucifera* seed oil (TNSO) was obtained from Durae Corporation (Gunpo, Korea). Bovine serum (BS) was purchased from Gibco (Grand Island, NY, USA). Fetal bovine serum (FBS) and Dulbecco’s modified Eagle’s medium (DMEM) were purchased from Hyclone (Logan, UT, USA). Penicillin, streptomycin and phosphate-buffered saline (PBS) were purchased from Capricorn (Ebsdorfergrund, Hesse, Germany). Dimethyl sulfoxide (DMSO), insulin, dexamethasone (DEX), 3-iso-butyl-1-methylxanthine (IBMX), 3,3′,5-Triiodo-L-thyronine (T3) and the other chemicals were purchased from Sigma-Aldrich (St. Louis, MO, USA).

### Cell culture and differentiation

Murine 3T3-L1 cells were provided by Prof. Barbara Corkey (Boston University School of Medicine, MA, USA). 3T3-L1 preadipocytes were seeded into 12-well plates at a density of 5 × 10^4^ cells/mL and cultured in growth media containing DMEM supplemented with BS (10% v/v), streptomycin (10 mg/mL) and penicillin (10,000 U/mL) at 37 °C in a humidified atmosphere of 5% CO_2_. Two days after the cells approached 100% confluency (day 0), differentiation was induced with an adipogenic cocktail (1 μM DEX, 0.5 mM IBMX, 1 μg/mL insulin, and 2 nM T3) added to basal medium (DMEM with FBS (10% v/v) and penicillin/streptomycin). On day 2, the first differentiation medium was replaced with the second differentiation medium consisting of the basal medium supplemented with 2 nM T3 and 1 μg/mL insulin. On day 4, the cells were further differentiated with the basal medium. TNSO was dissolved in DMSO and diluted with the culture medium to obtain the desired concentrations. TNSO was added on days 0, 2, and 4, while the control group was treated only with DMSO. The final concentration of DMSO was equal between the TNSO-treated and control groups.

### Measurement of cell viability

The viability of cultured cells was determined via the methylthiazolyldiphenyl tetrazolium bromide (MTT) method. Cells were cultured in 24-well plates at a density of 2.5 × 10^4^ cells/mL. After 24 h of TNSO treatment, 50 μL of 5 mg/mL MTT solution was added to each well and the cells were incubated for 4 h at 37 °C. Thereafter, 500 μL of DMSO was added to dissolve the formazan crystals. After 15 min, absorbance was measured at 590 nm using a microplate spectrophotometer (BioTek Instruments, Winooski, VT, USA).

### Oil Red O staining

On day 5, post-differentiation induction, 3T3-L1 cells were washed with PBS, fixed with 10% formalin solution for 20 min, and washed with 60% isopropyl alcohol. After drying completely, the cells were stained with 0.5% Oil Red O solution for 1 h at room temperature. Thereafter, stained cells were washed four times with distilled water, and photomicrographs were obtained using an Eclipse Ti2 inverted microscope (Nikon, Tokyo, Japan).

### Measurement of intracellular triglyceride levels

On day 5, post-differentiation induction, the medium was removed and 3T3-L1 cells were washed once with PBS. The cells were disrupted using lysis buffer (0.1% SDS, 100 mM Tris-HCl, and 1 mM EDTA) and the freeze-thaw cycle between − 80 °C and 37 °C was repeated three times. The intracellular triglyceride content was measured using a triglyceride determination kit (Sigma, St. Louis, MO, USA) in accordance with the manufacturer’s instructions. The absorbance was measured at 540 nm using a microplate spectrophotometer (BioTek Instruments, Winooski, VT, USA).

### Quantitative real-time PCR (qRT-PCR) analysis

To examine the effect of TNSO on adipogenic gene expression, 3T3-L1 preadipocytes were allowed to differentiate for 5 days with 0.75 μL/mL TNSO. TNSO was added on days 0, 2, and 4, whereas the control group was treated only with DMSO. On day 5, post-differentiation induction, total RNA of 3T3-L1 cells was extracted using the RNeasy Mini Kit (QIAGEN, Hilden, Germany). Target RNA was amplified using the AccuPower® GreenStar™ RT-qPCR Master Mix (Bioneer, Daejeon, Korea). Quantitative RT-PCR was performed on LightCycler® 96 system (Roche, Basel, Switzerland) as follows: reverse transcription at 60 °C for 15 min, pre-denaturation at 95 °C for 5 min, denaturation at 95 °C for 10 s, annealing at 55 °C for 30 s, and extension at 72 °C for 30 s. The denaturation step to extension step was cycled 45 times. Relative mRNA expression levels were normalized to those of β-actin as the internal control. The sequences of the primers were as follows: 5′-GAGGTATCCTGACCCTGA AGTA-3′ (β-actin forward), 5′-CACACGCAGCT CATTGTAGA-3′ (β-actin reverse), 5′-TGGAAGACAGCTCCTCCTCG-3′ (FABP4 forward), 5′-AATCCCCATTTACG CTGATGATC-3′ (FABP4 reverse), 5′-ACCTGGTAGACCACTGCATTGAC-3′ (FAS forward), 5′-CCTGATGAAACGACACATTCTCA-3′ (FAS reverse), 5′-TGAGACAG GAGATGTTGGAATG-3′ (adiponectin forward), 5′-ACGCTGAGCGATACACATA AG-3′ (adiponectin reverse).

### Measurement of PPARγ and STAT3 activation using *Gaussia* luciferase (GLuc) reporter cells

Stable 3T3-L1 PPARγ and STAT3 reporter cell lines were generated as follows. Briefly, consensus binding sites of PPARγ and STAT3 (AGGACAAAGGTCA for PPARγ and TTTCCGGGAA for STAT3) were identified using the TRANSFAC public database. The response element (RE) oligonucleotides, containing three consensus binding sequences, were cloned into the GLuc-DRE2-viral vector, wherein GLuc is regulated by a minimal promoter [14]. The expression of GLuc is induced when a target transcription factor binds to its consensus binding site. Thereafter, stable 3T3-L1 PPARγ and STAT3 reporter cell lines were generated through lentiviral transduction.

To profile the activation of PPARγ and STAT3, the reporter cells were differentiated in 12-well plates as described above. The supernatant was harvested on day 1, day 3, and day 5 post-differentiation induction and used to measure GLuc activity. GLuc activity (Relative Light Units; RLU) was assessed using the *Gaussia* Luciferase Flash Assay Kit (Thermo Fisher Scientific, Waltham, MA, USA) and TriStar^2^ LB 942 Modular Multimode Microplate Reader (Berthold technologies, Bad Wildbad, Germany) in accordance with the manufacturers’ instructions. After normalization with cell density, relative fold changes were determined at each time point.

### Statistical analysis

All tests were performed with two sets of triplicate experiments. Statistical comparisons between samples were performed using Student’s *t*-test.

## Results

### TNSO does not affect 3T3-L1 viability

**Fig. 1:**
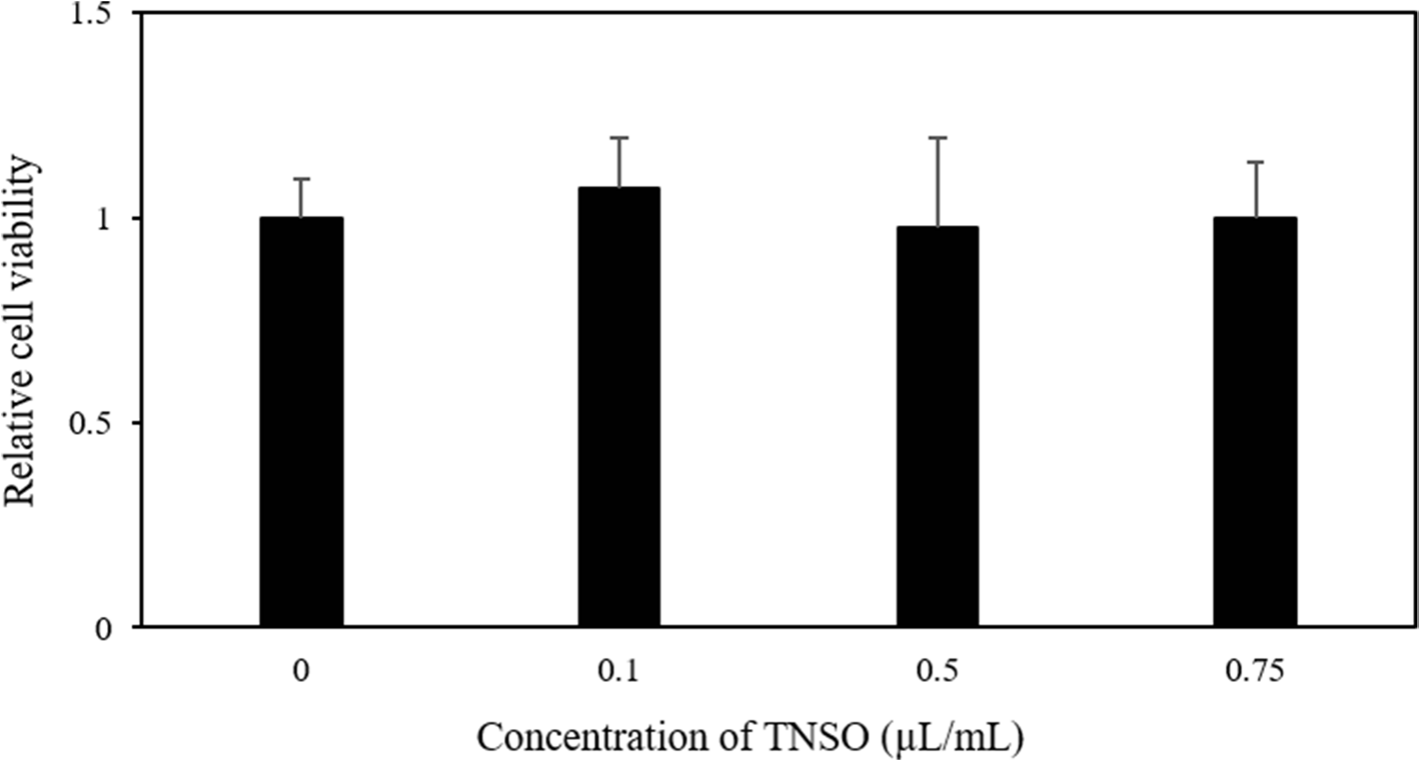
The effect of *Torreya nucifera* seed oil (TNSO) on cell of 3T3-L1 cells. Cytotoxicity of 3T3-L1 cells treated with TNSO for 24 h was determined using the methylthiazolyldiphenyl tetrazolium bromide assay. All tests were performed with two sets of triplicate experimental setups. Bars indicate mean and SEM

To determine the viability of 3T3-L1 cells upon treatment with TNSO, cells were incubated with various concentrations (0.1 - 0.75 μL/mL) of TNSO. As shown in Fig. 1, TNSO displayed no toxicity towards 3T3-L1 cells at all tested concentrations. Additionally, the potential toxic effect for another cell line was examined and TNSO treatment did not decrease the viability of RAW264.7 cells (Supplementary Fig. 1).

### TNSO increases lipid accumulation during 3T3-L1 adipocyte differentiation

**Fig. 2:**
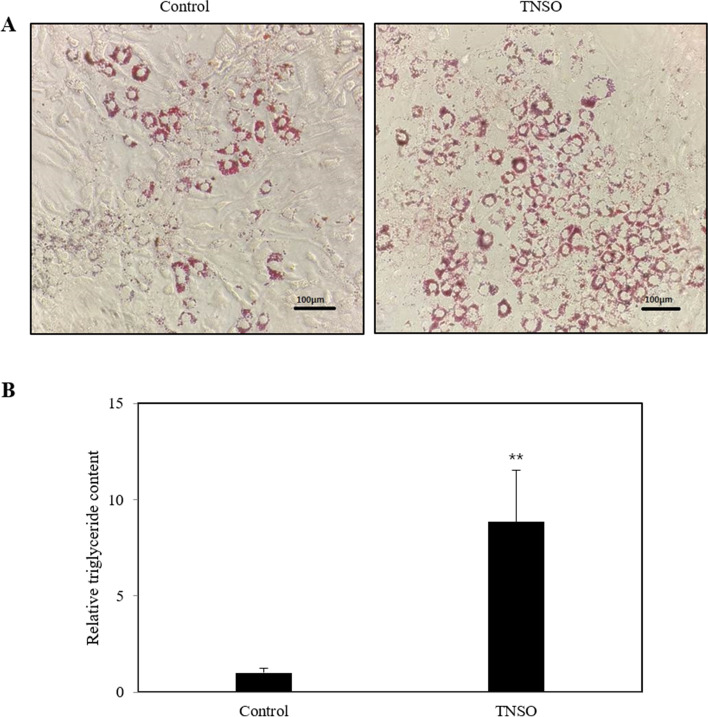
The effect of *Torreya nucifera* seed oil (TNSO) on lipid droplet formation and intracellular triglyceride contents in 3T3-L1 cells. 3T3-L1 preadipocytes were cultured for 5 days post-differentiation induction in medium supplemented with DMSO or TNSO dissolved in DMSO. **a** Representative images of 3T3-L1 adipocytes stained with Oil Red O. Scale bar = 100 μm **b** Intracellular triglyceride contents in 3T3-L1 adipocytes. All tests were performed in triplicate in two independent experiments. The bars indicate mean and SEM. **: *p* < 0.01 compared to the samples treated with DMSO

To examine the effect of TNSO on 3T3-L1 adipocyte differentiation, 3T3-L1 preadipocytes were allowed to differentiate for 5 days with 0.75 μL/mL TNSO. As shown by Oil Red O staining, TNSO treatment increased the number of stained lipid droplets (Fig. 2a). For quantitative comparison, we further performed a triglyceride determination assay to measure intracellular triglyceride levels. As a result, the triglyceride content of TNSO-treated cells was approximately 8.5-fold higher than that of the control group (Fig. 2b). These results suggest that TNSO treatment increases lipid accumulation, indicating the progression of adipocyte differentiation.

### TNSO upregulates adipogenic gene expression

**Fig. 3:**
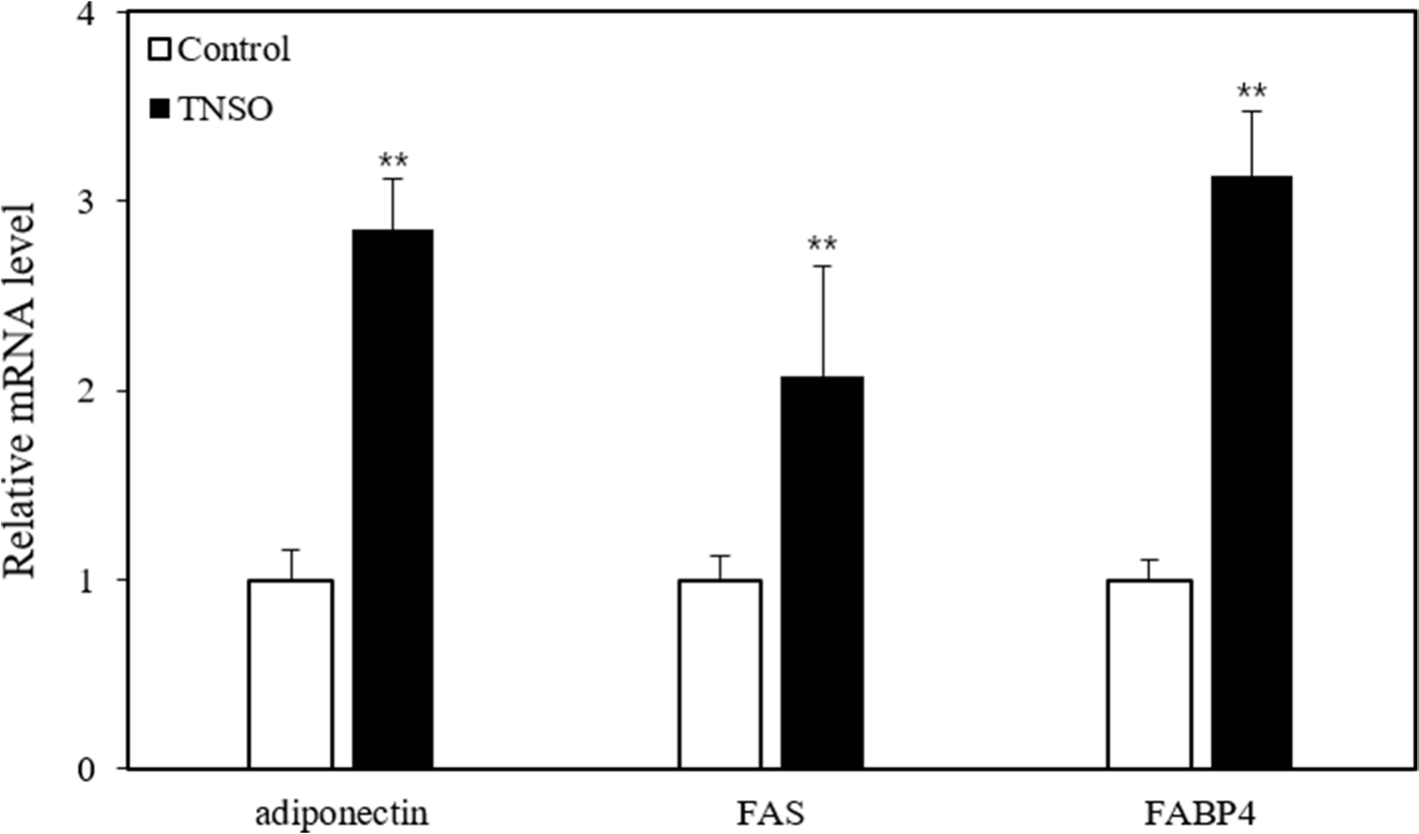
The effect of *Torreya nucifera* seed oil (TNSO) on the expression of adipogenic genes. Total RNA was extracted from 3T3-L1 adipocytes on day 5 post-differentiation induction. The mRNA levels of adiponectin, fatty acid synthase (FAS) and adipocyte fatty acid-binding protein 2 (FABP4) were determined through quantitative real-time PCR analysis and normalized with corresponding β-actin mRNA expression levels. All tests were performed in triplicate in two independent experiments. The bars indicate mean and SEM. **: *p* < 0.01 compared to samples treated with DMSO

To further investigate the effect of TNSO on 3T3-L1 adipocyte differentiation, we analyzed the mRNA expression levels of adipogenic genes including adiponectin, FABP4, and FAS on day 5 post-differentiation induction. Adiponectin, a specific adipokine produced by adipocytes, was upregulated by 2.6-fold with TNSO treatment. Furthermore, the levels of FABP4 and FAS, which play key roles in fatty acid metabolism, were significantly upregulated by 3.1-fold and 2.1-fold, respectively (Fig. 3). These results indicate that TNSO improves 3T3-L1 adipocyte differentiation by upregulating adipogenic gene expression.

### TNSO induces PPARγ and STAT3 activation

**Fig. 4:**
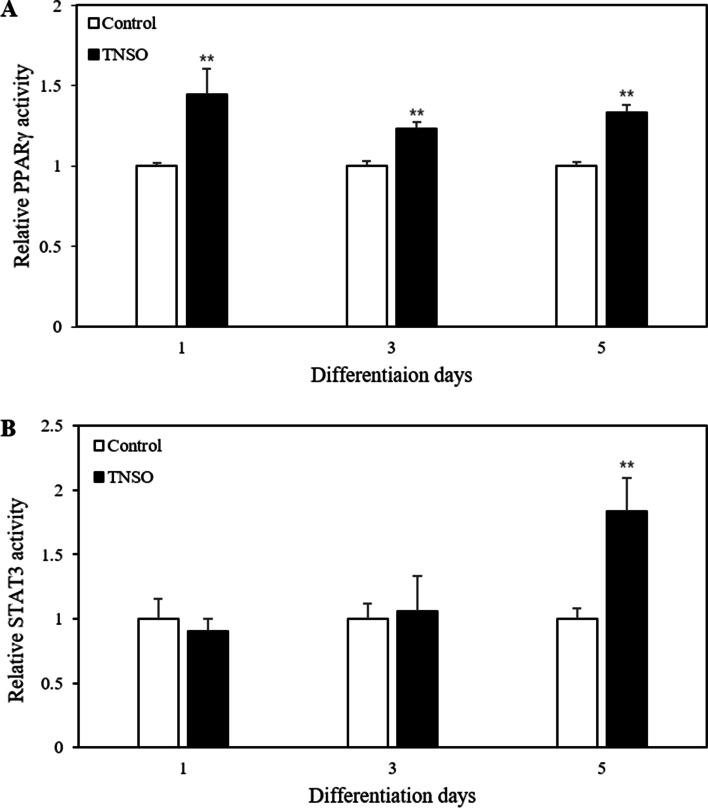
The effect of *Torreya nucifera* seed oil (TNSO) on the activation of PPARγ and STAT3. 3T3-L1 reporter cells for PPARγ or STAT3 were differentiated and supernatants containing secreted *Gaussia* luciferase (GLuc) were harvested on days 1, 3, and 5 post-differentiation induction. Relative activity of **a** PPARγ and **b** STAT3 was determined from the fold-change normalized with the activity of cells treated with DMSO at each time point. All tests were performed in triplicate in two independent experiments. The bars indicate mean and SEM. **: *p* < 0.01 compared to samples treated with DMSO

Adipocyte differentiation is a highly complex process involving morphological alteration and changes in the expression of several adipogenic genes [15]. Hence, it is necessary to investigate how TNSO affects the activation of transcription factors regulating the expression of adipogenic genes. Changes in transcription factor activity profiles were monitored using 3T3-L1 reporter cell lines, which report the activation of a target transcription factor through secreted *Gaussia* luciferase. PPARγ functions as a master regulator of adipogenic genes and STAT3 is reported to be involved in the early stage of 3T3-L1 adipocyte differentiation [16, 17]. In this study, 0.75 μL/mL TNSO treatment increased PPARγ activity by 44% on day 1 and a similar degree of increased activity was maintained until day 5 after the induction of differentiation (Fig. 4a). In the case of STAT3, a significant increase by 84% was detected on day 5 (Fig. 4b). These results suggest that TNSO-induced activation of PPARγ and STAT3 contributes to the improvement in adipocyte differentiation.

## Discussion

TNSO has been used as an edible oil and traditional medicine. Although studies on *Torreya nucifera* have previously shown anti-bacterial, anti-inflammatory and anti-viral activities, limited information has been available regarding its effects on adipocyte metabolism at the cellular level. Hence, this study aimed to investigate the effects of TNSO on 3T3-L1 adipocyte differentiation and the underlying mechanism. We found that the formation of lipid droplets and intracellular triglyceride content were increased upon TNSO treatment (Fig. 2). To confirm the stimulatory role of TNSO on adipocyte differentiation, we compared the expression level of genes reflecting characteristics of adipocyte differentiation. Adiponectin is a specific adipokine produced by adipocytes and exerts anti-apoptotic, anti-inflammatory and insulin-sensitive effects [9, 18]. FABP4 is a lipid-binding protein playing a key role in intracellular transport and metabolism of fatty acids [8, 19]. FAS is an enzyme that mediates fatty acid synthesis [7, 20]. In this study, adiponectin, FABP4 and FAS levels were upregulated upon TNSO treatment during 3T3-L1 adipocyte differentiation (Fig. 3).

Adipocyte differentiation is regulated in a complicated manner through the interaction of several transcription factors. A previous study reported a marked increase in the activity of major adipogenic transcription factors at the early stages of the adipocyte differentiation process [21, 22]. Hence, we monitored the activity of PPARγ and STAT3, which are closely associated with adipocyte differentiation, to elucidate the mechanism of action of TNSO. Consequently, PPARγ activity peaked upon TNSO treatment on day 1 and remained approximately 30% higher than that in the control group until day 5. Although STAT3 activity did not significantly change until day 3, it almost doubled on day 5 upon TNSO treatment. Deng et al. reported that induction of differentiation with an adipogenic cocktail led to an increase of STAT3 activity [5]. As the differentiation-inducing reagent is removed from day 4, the difference in STAT3 activation by TNSO starts to appear from day 5. Wang et al. showed that STAT3 inhibition suppressed adipocyte differentiation, suggesting that STAT3 signaling occurs upstream of PPARγ [23]. Because PPARγ activity increased from day 1, TNSO-derived PPARγ activation appeared to be independent of STAT3 activation. Numerous studies have reported that the expression of adiponectin, FAS and FABP4 is regulated by STAT3 and PPARγ [5, 7, 8]. Thus, the enhancement of adipocyte differentiation upon TNSO treatment can be explained with the increase in adipogenic gene expression by PPARγ and STAT3 activation.

Impaired adipocyte differentiation is associated with aging, oxidative stress and insulin resistance. As the levels of oxidative stress and insulin resistance increase due to reduced adipocyte renewal during aging, a strategy for improvement of adipocyte differentiation is needed. The present results suggest that TNSO may serve as a potential agent to treat diseases resulting from suppressed adipocyte differentiation.

## Conclusions

Thus, this study shows that TNSO significantly improves 3T3-L1 adipocyte differentiation by modulating PPARγ and STAT3 activity, concomitantly upregulating adipogenic gene expression and increasing lipid accumulation in adipocytes. These results establish the pro-adipogenic effect of TNSO and provide insights into its underlying mechanism of action at the cellular level. Therefore, our data supports that TNSO may be a promising agent for the recovery of impaired adipogenesis.

## Supplementary Information

**Additional file 1: Supplementary Fig. 1.** The effect of *Torreya nucifera* seed oil (TNSO) on viability of RAW264.7 cells.

## Data Availability

The raw data generated during the current study are available from the corresponding author upon request.

## Abbreviations

TNSO: *Torreya nucifera* seed oil
FAS: Fatty acid synthase
FABP4: Adipocyte fatty acid-binding protein
PPARγ: Peroxisome proliferator-activated receptor gamma
STAT3: Signal transducer and activator of transcription 3
